# Magnitude and associated factors of unmet need for family planning among reproductive-aged women in Ethiopia: An umbrella review

**DOI:** 10.1371/journal.pone.0308085

**Published:** 2024-08-01

**Authors:** Mulat Ayele, Gizachew Yilak, Abebaw Alamrew, Eyob Shitie Lake, Befkad Derese Tilahun

**Affiliations:** 1 Department of Midwifery, College of Health Science, Woldia University, Woldia, Ethiopia; 2 Department of Nursing, College of Health Science, Woldia University, Woldia, Ethiopia; Mount Sinai School of Medicine: Icahn School of Medicine at Mount Sinai, UNITED STATES OF AMERICA

## Abstract

**Introduction:**

The World Health Organization defines women with an unmet need for family planning as individuals who are fecund, sexually active, and wanting to either to limit childbirth or postpone their next birth but are not utilizing any form of contraception. Previous meta-analyses and systematic reviews have focused on this topic, but no comprehensive summary of the evidence was available. Therefore, the aim of this study was to provide a summary of the findings on the unmet need for family planning among reproductive-aged women in Ethiopia.

**Method:**

A search was conducted in PubMed, Google Scholar, CINAHL, Scopus, Cochrane Library, and RESEARCH 4 LIFE including Hinari, to identify relevant systematic reviews and meta-analyses of studies on the prevalence and determinants of the unmet need for family planning in Ethiopia. The methodological quality of the included studies was assessed using the Assessment of Multiple Systematic Reviews tool, and the estimates were pooled using a random-effects meta-analysis model.

**Results:**

The umbrella review included five studies with a total of 56,169 reproductive-aged women. The pooled prevalence of unmet need for family planning was found to be 27.44% (95% CI: 22.32–32.55), with the heterogeneity index (I square = 99.45%, P = 0.000). Failure to discuss with her partner about family planning methods (OR: 2.90, 95% CI: 1.81, 4.00), being illiterate (OR: 2.17, 95% CI: 1.42, 2.92), age at first marriage (OR: 1.37, 95% CI: 1.30, 1.44), and educational status of woman’s partner (OR: 2.61, 95% CI: 0.83, 4.40) were the factors significantly and positively associated with unmet needs for family planning.

**Conclusion:**

The study concludes that the unmet need for family planning in Ethiopia is a significant issue that can be addressed through increasing the educational levels of women and their partners, promoting open discussions about family planning, and preventing early marriages. Collaboration between families, school administrators, and healthcare providers is essential to mitigating this problem.

## Introduction

Family planning (FP) refers to a deliberate endeavor by a couple to anticipate, achieve, restrict, or regulate the desired number of children they wish to have through the use of contraceptive methods and addressing involuntary infertility issues. Contraception refers to the actions taken to prevent pregnancy. The indicators for achieving the sustainable development goal (SDG) 3.7., which aims to ensure universal access to sexual and reproductive healthcare services, including family planning, are based on the proportion of women of reproductive age who have their family planning needs satisfied using modern contraception methods [1, 2]. The World Health Organization (WHO) defines the unmet need for family planning as individuals who are capable of reproduction, engaged in sexual activity, but are not utilizing any type of contraception. These individuals express either a desire to refrain from having additional children or a wish to postpone their next child [3]. This factor serves as one of the measurements utilized to assess the attainment of comprehensive reproductive health coverage on a global scale [4]. Family planning represents a fundamental aspect of women’s rights and comprises the primary component of their reproductive health [5].

In order to ensure this entitlement, it is crucial to offer women and men unrestricted availability to free family planning service, including highly effective contraceptive methods. Family planning services play a critical role in preventing unintended pregnancies, which, when neglected or used inconsistently, contribute significantly to maternal mortality rates [6, 7]. Unplanned pregnancy has wide-ranging implications and represents a significant global public health concern that impacts individuals across all aspects of life. It is intricately connected to parental distress, induced abortions, infertility, adverse physical and mental health outcomes, social and economic repercussions, as well as increased risks of maternal and child mortality [8]. Family planning plays a pivotal role not only in directly enhancing reproductive health results but is also strongly correlated with advancements in health, education, and economic outcomes [9]. Due to its significance, this right has been integrated into the pursuit of universal access to reproductive health, which was a target under Sustainable Development Goal 3 aimed at enhancing maternal health. This integration signifies the required investments and anticipated advancements resulting from programmatic initiatives aimed at expanding the availability of effective contraceptive methods [4, 10]. Despite its significance, approximately 270 million women of reproductive age (between 15 and 49 years old) globally have a lack of access to contraception, indicating an unmet need in this critical aspect of reproductive health [11]. Sub-Saharan Africa (SSA) exhibits the highest prevalence of unmet need for contraception, with approximately one in five women facing a lack of access to contraception for the purpose of either spacing or limiting pregnancies [12].

The majority of developing countries are significantly behind in achieving the targets established by the SDGs pertaining to maternal mortality reduction and access to reproductive health services, which aimed to reduce maternal mortality by 75% and ensure universal access to reproductive health services by 2015. In many African nations, maternal mortality rates have actually shown an upward trajectory, contrary to the desired progress. Even in countries that have made some advancements, the target remains far from being attained [13, 14]. Various studies indicate that in certain Sub-Saharan African countries, a significant proportion of young women, ranging from 8% to 25%, discontinue their education as a result of unintended pregnancies stemming from unmet needs for family planning [6, 15]. Limited surveys conducted on the subject of unmet need for family planning in Ethiopia have indicated that unwanted pregnancies and unsafe abortions are significant factors contributing to maternal mortality [16].

The contraceptive prevalence rate in Ethiopia showed an increase from approximately 29% in 2011 to 36% in 2016 and further to 42%, with 41% using modern methods and1% using traditional methods in 2019 [16–18]. However, the level of unmet need for family planning did not decrease at the same rate as the contraceptive prevalence rate. In 2011, the unmet need was recorded at 25.3%, and it slightly decreased to 22% in 2016 [16, 17]. According to the 2016 Ethiopian Demographic and Health Survey (EDHS), a significant percentage of women in Ethiopia had an unmet need for family planning, with 22% facing this challenge. Among them, 13% expressed a need for spacing pregnancies, while 9% desired to limit their pregnancies but lacked access to appropriate family planning methods. The same report also highlighted that 58% of currently married women aged 15–49 expressed a desire for family planning in 2016. Out of these women, 36% were already using a contraceptive method, whether for spacing pregnancies (22%) or limiting births (14%) [17]. Even though both modern and traditional contraceptive methods were used in Ethiopia, the major source of modern contraceptive methods was public sectors, which accounts 87%. This indicates either there is no enough other alternative source or women not use other sources to met the demand of family planning [18]. The unmet need for family planning among currently married women aged 15–49 was found to be lowest in Addis Ababa (11%) and highest in the Oromia region (29%) [19].

The inconsistent nature of the report poses challenges for healthcare programs and clinical decision-making. Previous meta-analyses and systematic reviews have focused on this topic [20–24], but no comprehensive summary of the evidence was available for policy makers and clinicians to mitigate the factors of unmet need for family planning methods. Therefore, the aim of this study was to provide a summary of the findings on the unmet need for family planning and its determinant among reproductive-aged women in Ethiopia.

## Methods

This umbrella review was conducted using the methodology outlined in the umbrella review of systematic review and meta-analysis (SRMA) studies using Preferred Reporting Items for Systematic Review and Meta-Analysis (PRISMA) (S1 Checklist) and the Meta-analysis of Observational Studies guideline (MOOSE) [25–27]. These methodologies include comprehensive checklists consisting of 35 items that offer guidance for conducting and reporting observational studies with a high risk of bias and confounding, particularly when assessing retrospective data (S1 File). The review involved a systematic analysis of the included systematic review and meta-analysis studies on unmet need for family planning and its determinants in Ethiopia.

### Search strategy and source of information

We checked the PROSPERO databases (https://www.crd.york.ac.uk/prospero/) to see whether recently published or ongoing projects exist related to the topic to avoid any further unnecessary duplication. Thus, the findings revealed that there were no ongoing or published articles in the area of this topic. Then this umbrella review were registered in the PROSPERO databases with ID no. CRD42023482625. A comprehensive search about unmet need of family planning in Ethiopia was done through PubMed, Cochrane Library, research 4 life including Hinari, Google Scholar, CINAHL, and Scopus. The search was conducted from November 26 to 30, 2023. The search was conducted using PICO questions. All key terms were searched using a combination of the Boolean operators “AND” or “OR” as appropriate and the search was done by involving two independent researchers (BDT and MA), who reached a consensus without any disagreements (S2 File).

**Population**; reproductive-aged women

**Intervention (phenomena of Interest**); family planning

**Comparison;** met need for family planning

**Outcome;** the primary outcome of this review were the magnitude of unmet need family planning in Ethiopia and the second outcome was the factors associated with unmet need family planning in Ethiopia. Unmet needs for family planning is a proportion of women who are not pregnant and not postpartum amenorrhoeic and is considered fecund and want to postpone their next birth for 2 or more years or stop childbearing altogether but are not using a contraceptive method [17].

### Inclusion criteria

This umbrella review were include all systematic review and meta-analysis studies (SRMAS) which had a defined literature search strategy, appraised included studies using a relevant tool, and followed a standard approach in pooling studies and providing summary estimates.

### Exclusion criteria

The studies that were excluded lacked reporting on the measures of interest, were not in English, and included narrative reviews, editorials, correspondence, as well as studies with low methodological quality.

### Data extraction

Data from the included studies were extracted using a standardized data extraction form, developed in excel sheet. For each study, the following data were extracted: (1) identification data (first author’s last name and publication year), (2) measure of magnitude (prevalence for un meet need family planning) (3) factors associated with un meet family planning (odds ratio or relative risk) with 95% confidence intervals, (4) number of studies included, (5) total number of samples included, (6) publication bias assessment methods and scores, (7) quality assessment methods and scores, (8) data synthesis methods (random or fixed-effects model), and (9) the main conclusion of the study.

### Quality assessment

The quality of the studies was assessed using Assessment of Multiple Systematic Reviews (AMSTAR) checklist scores [28]. The checklist contains 11 indicators that are used to derive an overall score assessed as high quality (score > = 8), medium quality (score 4–7), and low quality (score < = 3). Three authors (MA, BD, and GY) assessed the quality of each study (i.e., methodological quality, sample selection, sample size, comparability and outcome, and statistical analysis of the study). In the case of disagreement between three authors, another two authors (ESL and AA) were involved and discussed and resolved the disagreement (S3 File).

### Data processing and analysis

The choice of the meta-analysis model was guided by the between studies heterogeneity, which was assessed by Higgins’s I2- Statistics [29]. According to Higgins et al. I2 < 49%, 50–75, and > 75% represents low, moderate, and high levels of heterogeneity, respectively. We intended to pool the estimates with fixed-effects models if the level of heterogeneity was < 50%. However, there was a high level of between-studies heterogeneity. Thus, the pooled prevalence estimates were calculated with the random-effects model, which accounts for both within-study and between-studies variations [30]. Due to the limited number of studies included in this umbrella review, we did not evaluate publication bias. To effectively assess the publication bias, a minimum of 10 studies is required [31]. Stata version 17.0 software was used for analyses.

## Result

### Literature search findings

**Fig 1:**
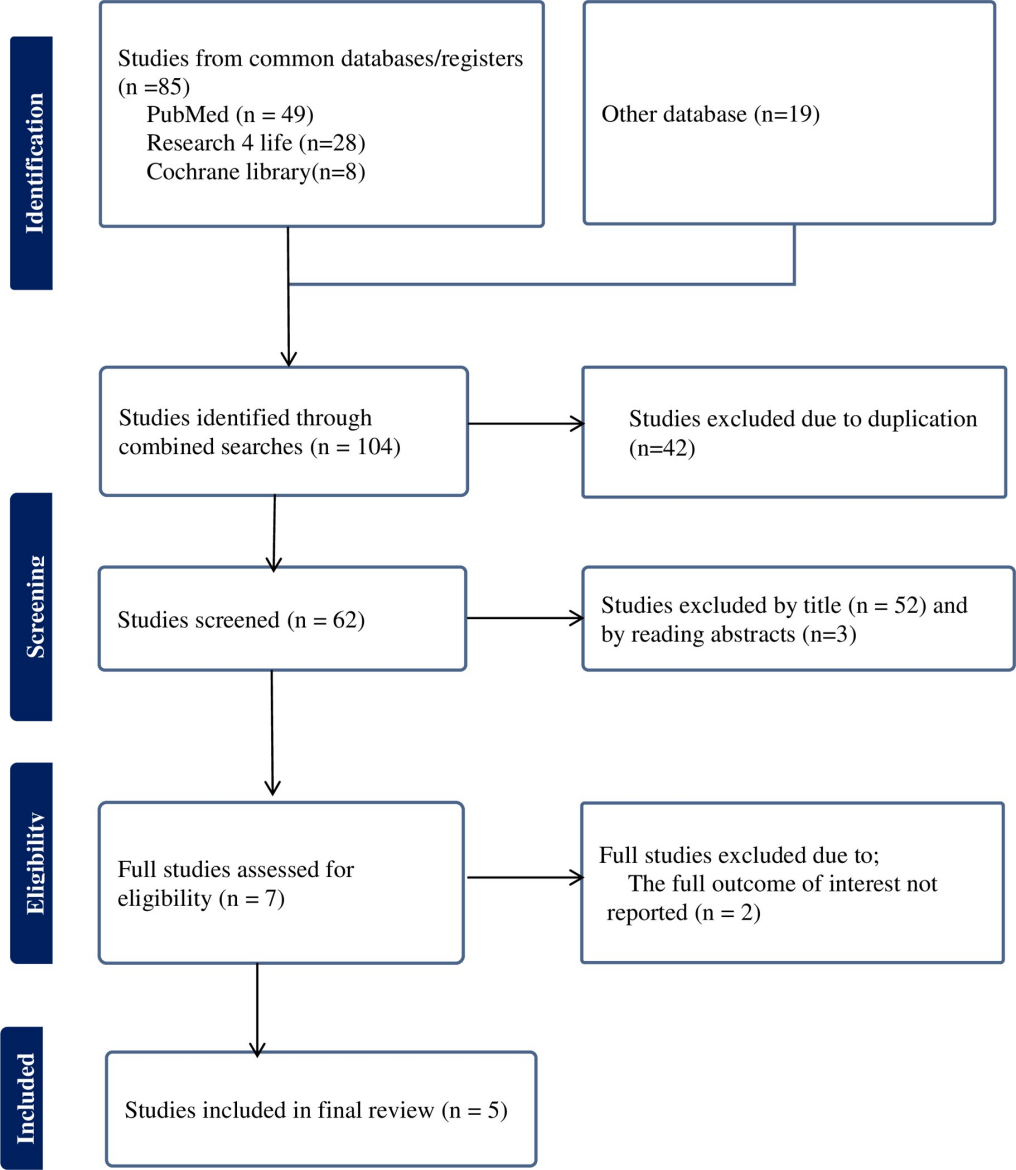
Flow chart of selection for umbrella review on unmet need for family planning among reproductive-aged women and its determinants in Ethiopia.

A comprehensive search of the database yielded a total of 104 articles. After removing duplicates, 62 articles remained. Further screening based on titles and abstracts led to the exclusion of 52 and 3 articles, respectively. The remaining articles underwent a full-text evaluation for inclusion criteria, resulting in the exclusion of an additional 2 papers. Finally,, a total of 5 studies [20–24]were included in this umbrella review (Fig 1).

### Characteristics of the included review studies

This umbrella review included five systematic review and meta-analysis [20–24]. These five SRMA studies incorporated a total of 50 primary cross-sectional studies, comprising a sample size of 56,169 women of reproductive age. The number of primary studies per SRMA ranges from 7 [23] to 15 [22]. Similarly, the sample size per each SRMA was ranged from 3333 [23] to 19,312 [21] reproductive-aged women. All included SRMA studies were assessed both the prevalence and factors of unmet need for family planning in Ethiopia. From those include reviews, two were done on reproductive-age women living with HIV [23, 24], which gives an insight for preventing unintended pregnancies among women living with HIV (prong 2). From the total of SRMA studies included in this umbrella review, two SRMA were published in 2020 [20, 22] and the other two in 2021 [23, 24]. However, the fifth study, Getahun DS et al. [21], were not published rather it was found as a preprint and we used it to reduce publication bias. Based on the included SRMA the prevalence of unmet need family planning were ranged from 19.99,(16.53–23.45), I^2^ = 43.0% [21] to 34.9(24.52–45.28), I^2^ = 98.9% [20] (Table 1).

**Table 1:** Characteristics of the included review studies on unmet need for family planning among reproductive-aged women in Ethiopia, 2023.

Authors (year)	Review objective	Search strategy	Population	Included studies	Sample size	Risk of bias	Reported prevalence	AMSTAR quality
Kefal B et.al (2021) [23]	To estimate the pooled prevalence unmet need for family planning in Ethiopia.	PubMed, Cochrane Library, Hinari, Google Scholar, CINAHL, and Global Health database. Search date was June 5–12, 2020, clear searching term was defined. All available studies from January 1, 2000, to June 1, 2020 were included. Clear inclusion and exclusion criteria were defined.	Reproductive-age women living with HIV	7, all cross sectional	3,333	The quality of included studies were appraised clearly	25.13 (19.97–30.29), I^2^ = 92.1%	10
Mekie M et al. (2021) [24]	To assess the level of unmet need for family planning and its predictors in Ethiopia.	PubMed, SCOPUS, EMBASE, HINARI, Google Scholar, and digital libraries of universities. Search date was July 25 to August 1/2020. Clear searching terms were defined. Both published and unpublished articles from January 2005 to August 1, 2020 were included. Had clear inclusion and exclusion criteria.	Reproductive-age women living with HIV	9, all were cross sectional.	6,154	The quality of included studies were appraised clearly using NOS	25.72(21.63–29.81), I2 = 94.3%.	9
Worku SA et al. (2020) [20]	To estimate the pooled prevalence of unmet need for family planning and its association to occupational status of women and discussion to her partner in Ethiopia.	PubMed, Cochrane Library, Google Scholar, CINAHL, and Embase. All published and unpublished articles up to March 2019 were included. Clear searching terms were defined. Searching date not stated. Had clear inclusion criteria but not exclusion criteria.	Reproductive-age women	9, all were cross sectional	9,785	The quality of included studies were appraised clearly	34.9(24.52–45.28), I^2^ = 98.9%	10
Getaneh T et al. (2020) [22]	To identify the predictors of unmet need for family planning in Ethiopia.	PubMed, Embase, CINAHL, Google Scholar, HINARI portal, and Cochrane Library. All studies conducted till June 10, 2019 was included. Clear searching terms were defined. Searching date not stated. Had clear inclusion criteria and exclusion criteria	Reproductive-age women	15, (2 were done in institution based and 13, were community.	17, 585	Joanna Briggs Institute Meta-Analysis of Statistics Assessment and Review Instrument (JBI-MAStARI) was applied for critical appraisal.	31.45(26.52–36.39), I^2^ = 88.0%	11
Getahun DS et al. (2020) [21]	To determine the pooled Magnitude and determinants of unmet need for family planning in Ethiopia.	Google Scholar, PubMed, Cochrane library, MIDLINE, EMBASE, and Ovid Maternity and Infant Care Databases. Clear searching terms were defined. Published articles starting from 2013to March 2020 were included. Search date was March to April 2020. Clear inclusion and exclusion criteria were defined.	Reproductive-age women	10, all were cross sectional.	19,312	The quality of included studies were appraised clearly using NOS	19.99,(16.53–23.45), I^2^ = 43.0%.	8

### Primary studies

There was an overlap of primary studies within each of the five SRMA included in this umbrella review. As indicated in the Table 1, there were 50 primary studies included in the SRMA. However, upon conducting a critical appraisal of the five SRMA, it was found that only 18 primary studies did not overlap (Table 2). Conversely, at least two primary studies were included in two or more SRMA. For instance, six studies [32–37] were included in both the review by Kefal B et.al [23] and Mekie M et al. [24]. Similarly, four primary studies [38–41] were included in the reviews conducted by Worku SA et al. [20], Getaneh T et al. [22] and Getahun DS et al. [21]. Additionally, three remaining primary studies [42–44] were included in both the reviews by both Worku SA et al. [20] and Getaneh T et al. [22], while one study was included in both the reviews by Getaneh T et al. [22], and Getahun DS et al [21] in the SRMA.

**Table 2:** Primary studies included in the systematic review and meta-analysis on unmet need for family planning among reproductive-aged women in Ethiopia.

Review studies	Primary studies													
	Abeje andMotbaynor, [32]	Abubeker FA et al [33]	Feyssa MD et al, [34]	Feyissa and Melka, [35]	Kassie MD et al, [36]	Zewdie Z et al, [37]	Genet et al. [42]	Gebre et al. [38]	Dejenu et al. [39]	Tegegn et al. [43]	Gebrecherkos et al. [40]	Mota et al. [44]	Worku et al. [41]	Chafo et al. [45]
Kefal B et.al [23]	#	#	#	#	#	#								
Mekie M et al. [24]	#	#	#	#	#	#								
Worku SA et al. [20]							#	#	#	#	#	#	#	
Getaneh T et al. [22]							#	#	#	#	#	#	#	#
Getahun DS et al. [21]								#	#		#		#	#

Note

# indicates number of primary studies included in each SRMA.

### Methodological quality of the included SRMA studies

The methodological quality evaluation of the included SRMA was conducted using the AMSTAR tool [28]. The quality score, which was measured on a scale of 11, ranged from 8 to 9, with an average score of 9.6 (S3 File).

### Prevalence of unmet need family planning among reproductive-aged women in Ethiopia

**Fig 2:**
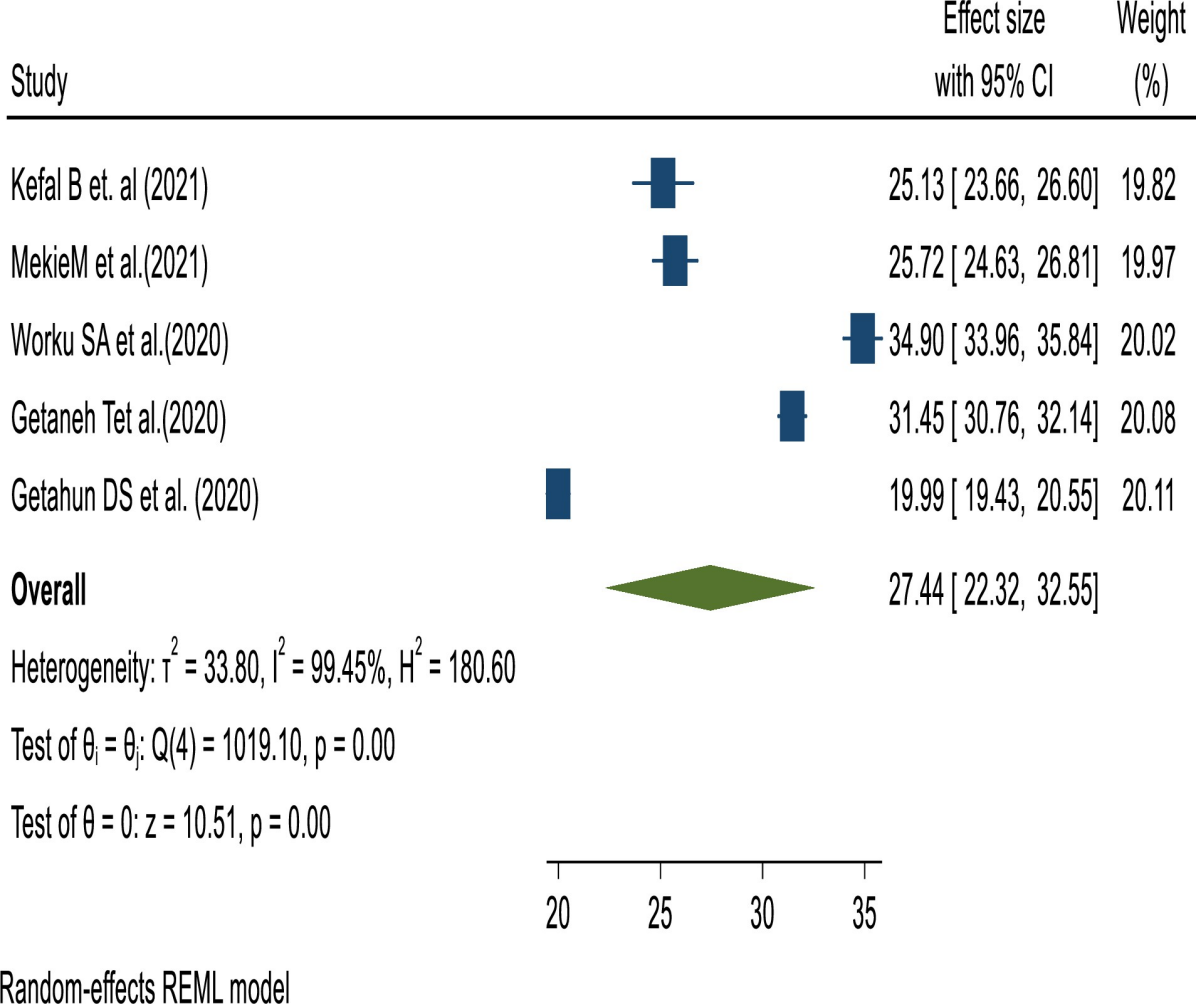
The pooled prevalence of unmet need for family planning among reproductive-aged women in Ethiopia.

The pooled prevalence of unmet need for family planning among reproductive-aged women in Ethiopia was 27.44%, (95% CI: 22.32–32.55), with the heterogeneity index (I2 = 99.45%, P = 0.000) showing substantial heterogeneity of different reviews (I2> 75%). Therefore, we have used the random effect model to resolve the issue of heterogeneity among the included reviews (Fig 2).

### Determinants of unmet need family planning among reproductive-aged women in Ethiopia

The included SRMA studies [20–24] in this umbrella review identified factors associated with unmet need for family planning. Those factors were, maternal age, maternal illiteracy, failure to discuss with her partner, lack of accesses information about family planning, being hose wife, age at first marriage, partner with no formal education, partner’s poor attitude towards family planning, poor knowledge about family planning and history of discussion with health care worker.

One systematic review and meta-analysis study [24] reported that, maternal age and accesses of information about family planning had a significant association with the prevalence of unmet need for family planning. Women whose age is 15–24 years were almost three times more likely to experience an unmet need for family planning compared to those with ages ≥ 35 years (OR = 3.12; 95% CI: 1.59, 6.11). According to this report, women who had no access to family planning information were 4.7 times more likely to experience unmet need for family planning compared to their counterparts (OR = 4.70; 95% CI: 2.83, 7.81). Moreover, one SRMA [20] showed that, maternal occupation had a significant association with unmet need for family planning. Accordingly, the odds of unmet need for family planning were 1.6 times higher among house wife as compared to women’s who have government employed (OR = 1.6, 95% CI: 1.29, 1.99). The other one SRMA [21] revealed that partners poor attitude towards family planning, poor knowledge about family planning and history of discussion with health care worker were the factors significantly associated with family planning. Accordingly, partners poor attitude towards family planning were 9 times more likely to get unmet need for family planning compared to good attitude (OR = 9.09; 95% CI: 3.85, 20). Similarly, women who had poor knowledge about family planning were 3.15 times more likely to experience unmet need for family planning than their counter parts (OR = 3.15; 95% CI: 1.75, 5.69). Additionally this SRMA also reported that women who had not history of discussion with health care worker were 3.2 times more likely to get unmet need for family planning than their counter parts (OR = 3.22; 95% CI: 1.47, 7.14).

**Fig 3:**
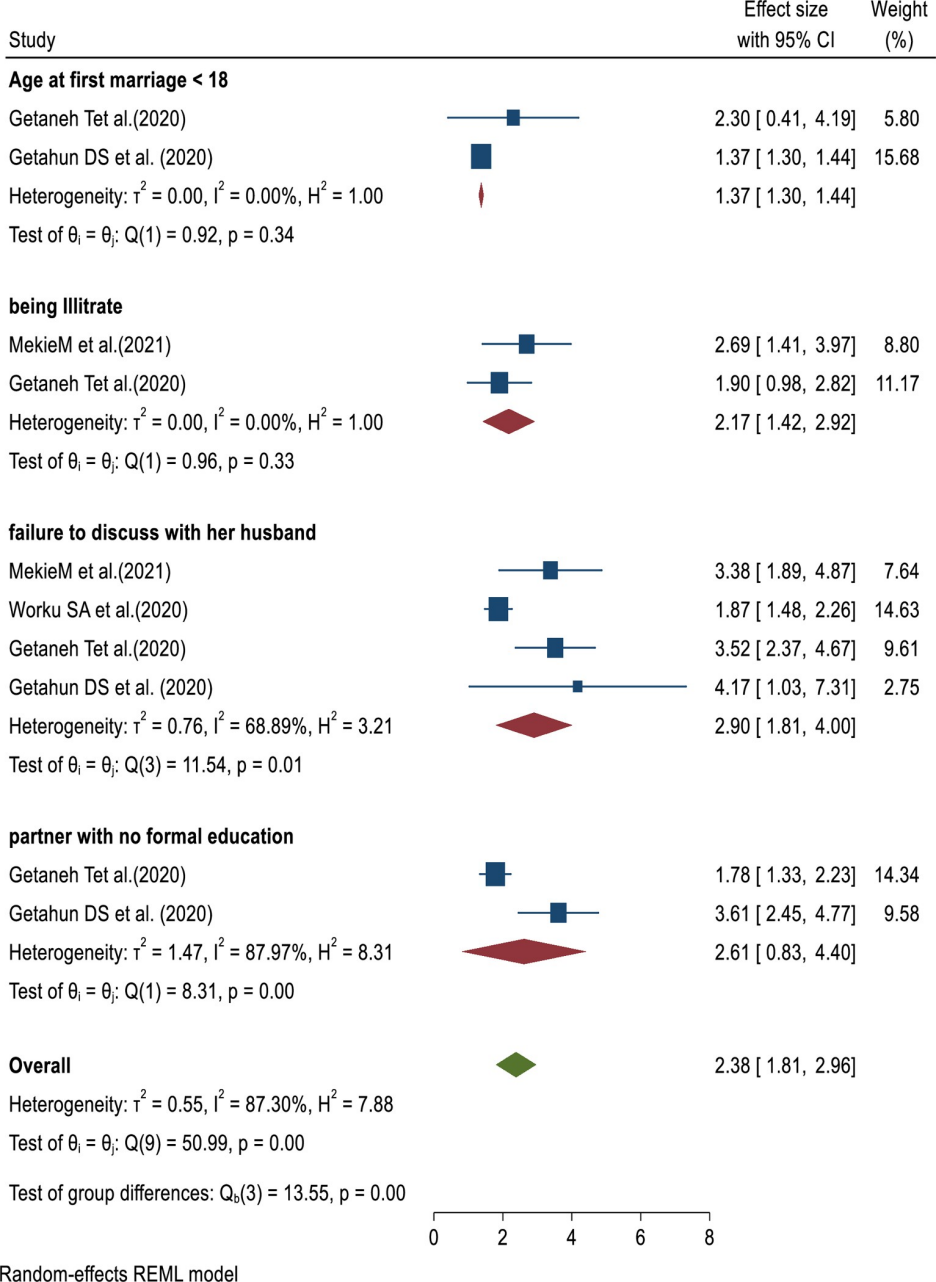
Umbrella review about the determinants of unmet need for family planning among reproductive-aged women in Ethiopia.

This umbrella review included four SRMA [20–22, 24] revealed that, there were a significant association between failure to discuss with her partner about family planning and unmet need for family planning. A woman who failed to discuss with her partner about family planning methods were 2.9 times more likely to experience unmet need for family planning than their counter parts (OR = 2.90, 95% CI: 1.81, 4.00) (Fig 3).

Furthermore, two additional SRMA [22, 24] reported that, there were an association between maternal educational level and unmet need for family planning. Being illiterate was 2.17 times more likely to get unmet need family planning than their counter parts (OR = 2.17, 95% CI: 1.42, 2.92) (Fig 3).

The other two SRMA [20, 22] revealed that, age at first marriage had a significant association with unmet need of family planning. Women whose age at first marriage less than 18 years were 1.37 times more likely experience unmet need family planning than their counter parts (OR: 1.37, 95% CI: 1.30, 1.44) (Fig 3).

Moreover, two SRM [21, 22] indicated a significant association between the educational status of women’s partners and unmet need for family planning. However, in this umbrella review, it was found that women whose partners did not have a formal education did not show a statistically significant association with unmet need for family planning (OR: 2.61, 95% CI: 0.83, 4.40) (Fig 3).

## Discussion

Although SRMA are highly regarded as evidence-based practices in health science clinical settings and programs, an umbrella review provides more comprehensive and informative insights when multiple SRMA have already been conducted on a specific research topic [46]. Therefore, this umbrella review aimed to summarize the findings of five existing systematic reviews and meta-analyses concerning the unmet need for family planning among reproductive-aged women and its determinants in Ethiopia.

The results of this umbrella review revealed that the overall pooled prevalence of unmet need for family planning in Ethiopia was 27.44% (CI: 22.32–32.55). This finding aligns with similar studies conducted in Bulgaria, where the prevalence was found to be 23% [47], as well as in Ecuador (25%) and Peru (29%) [48]. However, the prevalence of unmet need for family planning in this umbrella review was higher compared to the study conducted in southeast Nigeria, which reported a prevalence of 21.4% [49], in Iran,(2.6% [50], urban Cameroon (20.4%) [51] and the Dominican Republic, (21%) [48]. The higher prevalence in our country may be attributed to difficulties in accessing healthcare facilities due to long distances or limited availability, which makes obtaining contraception and family planning service extremely challenging. For instance, in Ethiopia, two in ten health care facilities offer any modern type of family planning service. Moreover, the availability of modern FP services is lower among clinics and health posts than other facility types [52]. Furthermore, a significant proportion (84%) of family planning methods in our country are obtained from government sources in exempted service and given by health extension workers, nurses, midwives, and doctors in health facilities [19]. Therefore, by enhancing the presence of healthcare facilities such as health posts and centers in rural regions, we can enhance the accessibility of family planning methods and consequently improve the fulfillment of family planning requirements in our nation. Furthermore, the involvement of health extension workers in rural communities serves to reinforce the distribution of family planning methods among women, ultimately reducing the unmet need for family planning in our country. On the other hand, the prevalence found in this umbrella review is lower than the study conducted in the North West Region of Cameroon, which reported a prevalence of 46.6% [53], as well as in India (55.3%) [54]. The differences observed could be due to variations in sample sizes, study areas, and the implementation of government policies. Our findings provide a comprehensive assessment of the overall unmet need for family planning in the entire country, whereas the listed studies were single studies limited to specific territories within the country. Additionally, the differences in prevalence may be influenced by factors such as varying levels of education, knowledge about contraceptives, fertility desires, and increased demand for family planning methods, particularly in India [55]. Enhancing women’s empowerment and educational attainment leads to heightened awareness among women regarding family planning methods, as well as their associated benefits and potential complications. Consequently, it becomes imperative to prioritize the advancement of women’s education levels in Ethiopia, as this will further encourage their understanding and utilization of family planning methods.

The umbrella review also found factors that were significantly associated with unmet need for family planning among women of reproductive age. Specifically, women who did not discuss family planning methods with their partners were 2.9 times more likely to experience unmet need for family planning compared to women who had such discussions. This finding is supported by a study conducted in the urban area of Cameron [51], North West Region, Cameroon [53]. One possible explanation for this is that men generally have a lower unmet need for family planning compared to women because they often desire to have more children, or have them sooner. This is evidenced as, among currently married women aged 15–49 who are not using a family planning method, 10% reported that their decision was influenced by their husband. Additionally, husband disapproval was identified as one of the reasons for contraceptive or family planning discontinuation [19]. Therefore, when women discuss family planning with their husbands, it reduces the likelihood of their husbands disapproving or retaliating against the use of family planning [56]. For instance, among currently married women aged 15–49 who are using a family planning method, 73% reported making the decision jointly with their husbands. This indicates that through discussion, any disagreements about the need for family planning and the desire for children were resolved [19].

Although the educational status of women’s partners did not show a significant association with unmet need for family planning in this umbrella review, two systematic reviews and meta-analyses (SRMAs) [21, 22] included in this review indicated that illiteracy among women’s partners was significantly associated with unmet need for family planning. Similarly, the educational level of women themselves was also significantly associated with unmet need for family planning. Illiterate women were 2.17 times more likely to experience unmet need for family planning compared to their counterparts. This finding was supported by various studies [19, 57–59]. This might be due to the fact that illiteracy is associated with a lack of knowledge about the importance of family planning in reducing unwanted pregnancies, unsafe abortions, birth spacing, and its role in decreasing maternal and neonatal morbidity and mortality [56]. Furthermore, as the level of education increases, the need for using family planning also increases. The use of modern contraceptives among currently married women rises from 31% for women with no education to 51% for women with secondary education or higher [19]. Therefore, empowering women and improving their level of education are crucial strategies for enhancing and promoting sexual and reproductive health, including family planning.

Regarding age at first marriage, women who got married before the age of 18 were 1.37 times more likely to experience unmet need for family planning compared to women who married at a later age. This finding was supported by a study conducted in eastern Sudan [60] and urban slums of Lucknow [61]. One possible explanation for this is that younger women, particularly those in their teenage years or below 18, are more influenced by their families, intimate partners, and peer pressure, which can impact their reproductive health needs and decision-making, including the need of family planning [62]. Similarly, women between the ages of 15–24 were nearly three times more likely to have unmet need for family planning compared to those who were 35 years or older [24]. This finding was concurred with a study done in Nigeria [63]. This is due to the higher level of unmet need for family planning among young women may be attributed to poor access to and low awareness of where and how to obtain family planning services.

Concerning to women’s occupation, being house wife is also significantly associated with unmet need family planning. This finding aligns with a study conducted in eastern Sudan [60]. The reason behind is that housewives often have lower levels of education compared to women in other occupations. This lower educational attainment can lead to a lack of awareness and understanding about family planning methods, their benefits, and how to access them. As a result, they may have inadequate knowledge about contraceptive options and their effective use. Moreover, housewives may have limited decision-making power and autonomy within their households. They may face resistance or disapproval from their husbands or other family members regarding the use of family planning methods. This lack of control and influence over reproductive choices can contribute to an unmet need for family planning [17]. Furthermore, women who had poor knowledge about family planning were 3.15 times more likely to experience unmet need for family planning than their counter parts. This finding is supported by a study done in Chidambaram, India [64]. The reason behind this association could be that women with inadequate knowledge about the advantages, disadvantages, and complications of not using family planning methods may delay their decision to utilize family planning services. This delay contributes to a higher prevalence of unmet need for family planning [17]. Similarly, partners who had poor attitude towards family planning were 9 times more likely to get unmet need family planning compared to those who had good attitude. This result is congruent with a study done in Uganda [65]. The reason behind this association may be attributed to the significant role of male involvement in the utilization of family planning services. Husbands with unfavorable attitudes towards family planning can influence the decision-making process and usage of family planning methods, thereby impacting the prevalence of unmet need for family planning [17]. Hence, it is crucial for healthcare providers and health extension workers to provide comprehensive health education regarding various family planning methods, including the advantages and complications associated with not using them. This educational effort should target women, their partners, and the entire community. By increasing knowledge and influencing attitudes towards family planning methods, we can effectively reduce the unmet demand for such methods in Ethiopia. Moreover, women who had not history of discussion with health care worker were 3.2 times more likely to get unmet need for family planning than their counter parts. This is consistent with a study done in Uttar Pradesh, India [66]. This is due to the fact that women who failed to discuss with healthcare providers lack awareness about the available options for family planning. As a result, rumors and perceived risks may increase, leading to a higher prevalence of unmet need for family planning. It is worth noting that 73% of women between the ages of 15 and 49 who are not using any contraceptive method reported that they did not have discussions about family planning with either a fieldworker or at a healthcare facility within the 12 months preceding the survey [17].

### Implication of the study

This study presents up-to-date and concise evidence on the unmet need for family planning in Ethiopia. It offers valuable insights for families, teachers, the Ministry of Education, and the Ministry of Women and Social Affairs regarding the impact of women’s education levels, women’s empowerment, and teenage pregnancy on the utilization of family planning methods and maternal and child health in Ethiopia. The study also holds implications for improving maternal and child health by reducing the unmet demand for family planning. It identifies factors that influence the fulfillment of family planning needs and proposes potential solutions in Ethiopia, such as enhancing the availability of family planning methods, promoting discussions between clients and healthcare providers particularly health extension workers to mitigate rumors towards family planning in the rural or low educated communities, and involving husbands in the decision-making process. By highlighting the gaps that require future focus; this contributes to the attainment of the goals and targets outlined in the Health Sector Transformation Plan II of the Ethiopian Ministry of Health. It serves as a valuable tool in guiding our efforts towards achieving these targets. Ultimately, the study guides efforts to increase knowledge and improve attitudes towards family planning among women and their husbands. It emphasizes the importance of providing health education and comprehensive counseling on family planning methods, including the potential maternal and child health complications that may arise from not using them. These interventions contribute to a decrease in the unmet demand for family planning in Ethiopia.

### Strength and limitation of the study

We made extensive efforts to reduce the risk of bias by conducting thorough searches across multiple databases and involving two independent researchers who reached a consensus without any disagreements. As far as we are aware, no comprehensive assessment in the form of an umbrella review has been conducted on the topic of unmet need for family planning in Ethiopia, despite the availability of various empirical studies and specific SRMA studies. Additionally, we examined the primary research within the SRMA reports to identify any overlapping data among the included SRMA studies. Although we have taken steps to minimize or address potential limitations, the absence of similar reviews conducted in other countries presents a challenge when directly comparing our findings with those of other studies. As a result, we primarily rely on making comparisons with individual primary studies in order to draw meaningful conclusions. This research exclusively evaluates quantitative studies; therefore, future researchers should incorporate qualitative research methods to gain deeper understanding of the factors influencing unmet need for family planning.

## Conclusion

The prevalence of unmet family planning needs in Ethiopia remains high. Factors contributing to this issue include maternal age, maternal illiteracy, lack of communication with partners about family planning, limited access to information, being a housewife, early marriage, and partner’s lack of education, negative attitude towards family planning, insufficient knowledge, and lack of discussions with healthcare workers. To address this, families, schools, governments, and the community leaders should promote women’s education and independence. This will empower women, improve access to family planning information through media and healthcare providers, and encourage open discussions between couples regarding sexual and reproductive health. Efforts should also be made to discourage early marriages by family, community leaders and government, as they impact the need for and decision-making towards family planning. Healthcare providers should engage in discussions with clients about family planning methods and their potential complications in case they are not utilized. Collaboration among government agencies, NGOs, and community organizations is necessary to implement the recommendation, which improves or met the family planning service need and utilization. The government should actively oversee and assess the efficiency of family planning programs, specifically in terms of raising awareness, providing counseling on different methods, implementing a women-friendly care approach, and promoting open discussions between individuals and healthcare providers.

## Supporting information

S1 File MOOSE checklist. (DOCX)

S2 File Search strategy. (DOCX)

S3 File Quality assessment. (DOCX)

S1 Checklist PRISMA 2020 checklist. (DOCX)

## Abbreviation

AMSTAR: Assessment of Multiple Systematic Reviews
EDHS: Ethiopian Demographic Health Survey
FP: Family Planning
SDG: Sustainable Development Goal
SRMA: Systematic Review and Meta-Analysis
WHO: World Health Organization
