# Supplementary material for: Magnitude and associated factors of unmet need for family planning among reproductive-aged women in Ethiopia: An umbrella review
Source: PLoS One. 2024 Aug 1;19(8):e0308085. doi: 10.1371/journal.pone.0308085 (PMC11293703; doi:10.1371/journal.pone.0308085)
Supplement: S1 File — (DOCX) [file pone.0308085.s001.docx]

S 1 file; MOOSE cheklist on unmet need for family planning among reproductive aged women in ethiopia

| Criteria | | Brief description of how the criteria were handled in  the meta-analysis |
| --- | --- | --- |
| **Reporting of background should**  **include** | |  |
| 1 | Problem definition | Page 3: Unmet needs for family planning (FP) as individuals who are capable of reproduction, engaged in sexual activity, but are not utilizing any form of contraception. Previous meta-analyses and systematic reviews have focused on this topic, but no comprehensive summary of the evidence was available. |
|  | Hypothesis statement | Page:3 The contraceptive prevalence rate in Ethiopia showed an increase from approximately 29% in 2011 to 36% in 2016 and further to 41% in 2019. However, the level of unmet need for family planning did not decrease at the same rate as the contraceptive prevalence rate. In 2011, the unmet need was recorded at 25.3%, and it slightly decreased to 22% in 2016. |
|  | Description of study outcomes | Page 5: Unmet needs for family planning is a proportion of women who are not pregnant and not postpartum amenorrhoeic and is considered fecund and want to postpone their next birth for 2 or more years or stop childbearing altogether but are not using a contraceptive method |
|  | Type of exposure or intervention used | Page 5: family planning |
|  | Type of study designs used | Page 4: Umbrella review |
|  | Study population | Page 5: reproductive aged women |
| **Reporting of search strategy should include** | |  |
|  | Qualifications of searchers | Page 5: The credentials of the two investigators  BDT and MA are indicated in the author list. |
|  | Search strategy, including time period included in the synthesis and keywords | Page 4-5: The search was conducted from November 26 to 30, 2023. Key words include; unmet need family planning, unmet need. |
|  | Databases and registries searched | Page 4: PubMed, Cochrane Library, research 4 life including Hinari, Google Scholar, CINAHL, and Scopus. |
|  | Search software used, name and version, including special features | Page 4: We did not employ search software. Mendeley  was used to merge retrieved citations and eliminate  Duplications. |
|  | Use of hand searching | Pages 5: We hand-searched bibliographies of retrieved papers for additional references |
|  | List of citations located and those excluded, including justifications | Pages 4-5: Details of the literature search process are outlined in supplementary file. |
|  | Method of addressing articles published in languages other than English | Page 5: We limited to the studies published in English |
|  | Method of handling abstracts and unpublished studies | Page 4: We have contacted authors for abstracts and unpublished studies through email. |
|  | Description of any contact with authors | Page 4: They did not respond |
| **Reporting of methods should include** | |  |
|  | Description of relevance or appropriateness of studies assembled for assessing the hypothesis to be tested | Pages 5: Detailed inclusion and exclusion criteria were described in the study selection section. |
|  | Rationale for the selection and coding of data | Page 5- 6: Data extracted from each of the studies were relevant to the first author, study characteristics, characteristics of participants, outcome characteristics. |
|  | Assessment of confounding | Not applicable |
|  | Assessment of study quality, including blinding of quality assessors; stratification or regression on possible predictors of study results | Page 6: The quality of the studies was assessed using Assessment of Multiple Systematic Reviews (AMSTAR) checklist scores. |
|  | Assessment of heterogeneity | Page 6: The between studies heterogeneity, which was assessed by Higgins’s I2- Statistics. According to Higgins et al. I2 < 49%, 50–75, and > 75% represents low, moderate, and high levels of heterogeneity, respectively |
|  | Description of statistical methods in sufficient detail to be replicated | Pages 6: Description of methods of meta-analyses was detailed in the data synthesis and analysis section. |
|  | Provision of appropriate tables and graphics | We included 1 flow chart, 3 summary tables and 5  Figures |
| **Reporting of results should include** | |  |
|  | Graph summarizing individual study estimates and overall estimate | Figure 2 |
|  | Table giving descriptive information for each study included | Table 1 |
|  | Results of sensitivity testing | Not applicable |
|  | Indication of statistical uncertainty of finding | Pages 12-13: 95% confidence intervals were presented with all summary estimates. |
| **Reporting of discussion should include** | | |
|  | Quantitative assessment of bias | Not applicable since studies were less than 10. |
|  | Justification for exclusion | Pages 5: Papers were excluded on the basis of exclusion criteria listed. |
|  | Assessment of quality of included studies | AMSTAR checklist scores was used to assess the quality. For detail see table 3. |
| **Reporting of conclusions should include** | |  |
|  | Consideration of alternative explanations for observed results | Page 17: We discussed the limitations of this study. |
|  | Generalization of the conclusions | Page 17: The prevalence of unmet family planning needs in Ethiopia remains high. Factors contributing to this issue include maternal age, maternal illiteracy, lack of communication with partners about family planning, limited access to information, being a housewife, early marriage, partner's lack of education, negative attitude towards family planning, insufficient knowledge, and lack of discussions with healthcare workers |
|  | Guidelines for future research | We recommend future studies on comparing the level of unmet need for family planning between married and unmarried women. |
|  | Disclosure of funding source | No funding source |
