# Supplementary material for: Magnitude and associated factors of unmet need for family planning among reproductive-aged women in Ethiopia: An umbrella review
Source: PLoS One. 2024 Aug 1;19(8):e0308085. doi: 10.1371/journal.pone.0308085 (PMC11293703; doi:10.1371/journal.pone.0308085)
Supplement: S2 File — (DOCX) [file pone.0308085.s002.docx]

S2 file: Search Strategy in different databases for unmet need for family planning among reproductive aged women in Ethiopia.

| Data base | Search | Query | Search results* |
| --- | --- | --- | --- |
| PubMed | #1 | Search:  (((((((((((prevalence) OR (proportion)) OR (incidence)) OR (epidemiology)) AND (determinants)) OR (factors)) OR (associated factors)) ) AND ((unmet need family planning))) OR (unmet need)) OR (family planning)) AND (Ethiopia) Filters: Meta-Analysis, Systematic Review | 53 |
|  | #2 | Search: (((((((((((((((((((prevalence) OR (proportion)) OR (magnitude)) OR (incidence)) AND (factors)) OR (determinants)) OR (predictors)) OR (factors associated)) OR (associated factors)) OR (risk factors)) AND (family planning)) OR (family planning utilization)) OR (family planning use)) OR (contraceptive use)) OR (contraceptive utilization)) OR (contraception)) AND (women)) OR (reproductive age women)) AND (systematic review)) OR (meta-analysis)) AND (Ethiopia) Filters: Meta-Analysis, Systematic Review | 1480 |
|  | #3 | Search: (((((((((((((prevalence) OR (proportion)) OR (incidence)) OR (epidemiology)) AND (determinants)) OR (factors)) OR (associated factors)) ) AND ((unmet need family planning))) OR (unmet need)) OR (family planning)) AND (Ethiopia)) AND (systematic review)) OR (meta-analysis) Filters: Meta-Analysis, Systematic Review | 225548 |
|  | #4 | Search: ((((((((((((((prevalence) OR (proportion)) OR (incidence)) OR (epidemiology)) AND (determinants)) OR (factors)) OR (associated factors)) ) AND ((unmet need family planning))) OR (unmet need)) OR (family planning)) AND (systematic review)) OR (meta-analysis)) AND (Ethiopia) Filters: Meta-Analysis, Systematic Review | 1474 |
|  | #5 | #1 AND #2 AND #3AND#4 | 49 |
| Research 4 life | #1 | prevalence and factors of unmet need family planning in Ethiopia systematic review and meta-analysis | 28 |
| Cochrane library | #1 | Cochrane Reviews matching prevalence in Title Abstract Keyword OR proportion in Title Abstract Keyword AND determinants in Title Abstract Keyword OR factors in Title Abstract Keyword AND unmet need family planning in Ethiopia in Title Abstract Keyword - (Word variations have been searched) | 8 |
| Other data base | #1 | prevalence and factors of unmet need family planning in Ethiopia systematic review and meta-analysis | 19 |

**= Date of search: from November 26 to 30, 2023.*
