# Supplementary material for: Magnitude and associated factors of unmet need for family planning among reproductive-aged women in Ethiopia: An umbrella review
Source: PLoS One. 2024 Aug 1;19(8):e0308085. doi: 10.1371/journal.pone.0308085 (PMC11293703; doi:10.1371/journal.pone.0308085)
Supplement: S3 File — (DOCX) [file pone.0308085.s003.docx]

S3 file; Methodological quality of the included studies about unmet need for family planning among reproductive aged women in Ethiopia based on the AMSTAR tool

| Authors (year) | Q1 | Q2 | Q3 | Q4 | Q5 | Q6 | Q7 | Q8 | Q9 | Q10 | Q11 | Total |
| --- | --- | --- | --- | --- | --- | --- | --- | --- | --- | --- | --- | --- |
| Kefal B et.al (2021)[21] | yes | yes | yes | yes | yes | Yes | yes | yes | yes | yes | no | 10 |
| MekieM et al.(2021)[22] | yes | no | yes | yes | yes | Yes | yes | no | yes | yes | yes | 9 |
| Worku SA et al.(2020)[18] | yes | yes | yes | yes | yes | Yes | yes | no | yes | yes | yes | 10 |
| Getaneh T et al.(2020)[20] | yes | yes | yes | yes | yes | Yes | yes | yes | yes | yes | yes | 11 |
| Getahun DS et al. (2020)[19] | yes | yes | yes | yes | yes | Yes | yes | no | no | no | yes | 8 |

*AMSTAR (Assessment of Multiple Systematic Reviews)*

*Q1: A priori design; Q2: Duplicate study selection and data extraction; Q3: Search comprehensiveness; Q4: Inclusion of grey literature; Q5: Included and excluded studies provided; Q6: Characteristics of the included studies provided; Q7: Scientific quality of the primary studies assessed and documented; Q8: Scientific quality of included studies used appropriately in formulating conclusions; Q9: Appropriateness of methods used to combine studies’ findings; Q10: Likelihood of publication bias was assessed; Q11: Conflict of interest – potential sources of support were clearly acknowledged in both the systematic review and the included studies.*
